# Antifungal potentiality of mycogenic silver nanoparticles capped with chitosan produced by endophytic *Amesia atrobrunnea*

**DOI:** 10.1016/j.sjbs.2023.103746

**Published:** 2023-08-05

**Authors:** Samiyah Saeed Al-Zahrani, Saleh Mohammed Al-Garni

**Affiliations:** a Department of Biology, Faculty of Science, King Abdulaziz University, Jeddah, Saudi Arabia; b Department of Biology, Faculty of Arts and Science, Albaha University, Albaha, Saudi Arabia

**Keywords:** Silver nanoparticles, Endophytic fungi, Nanocomposite, Chitosan, Capping agent

## Abstract

This research reports the fabrication of silver nanoparticles (AgNPs) from endophytic fungus, *Amesia atrobrunnea* isolated from *Ziziphus spina-christi* (L.). Influencing factors for instance, thermal degree of incubation, media, pH, and silver nitrate (AgNO_3_) molarity were optimized. Then, the AgNPs were encapsulated with chitosan (Ch-AgNPs) under microwave heating at 650 W for 90 s. Characterization of nanoparticles was performed via UV–visible (UV–vis) spectrophotometer, Fourier-transform infrared spectrophotometer (FTIR), zeta potential using dynamic-light scattering (DLS), and field-emission-scanning electron microscope (FE-SEM). Anti-fungal activity of Ch-AgNPs at (50, 25, 12.5, 6.25 mg/L) was tested against *Fusarium oxysporum*, *Curvularia lunata*, and *Aspergillus niger *using the mycelial growth inhibition method (MGI). Results indicated that Czapek-dox broth (CDB) with 1 mM AgNO_3_, an acidic pH, and a temperature of 25–30 °C were the optimum for AgNPs synthesis. (UV–vis) showed the highest peak at 435 nm, whereas Ch-AgNPs showed one peak for AgNPs at 405 nm and another peak for chitosan at 230 nm. FTIR analysis confirmed that the capping agent chitosan was successfully incorporated and interacted with the AgNPs through amide functionalities. Z-potential was −19.7 mV for AgNPs and 38.9 mV for Ch-AgNPs, which confirmed the significant stability enhancement after capping. FES-SEM showed spherical AgNPs and a reduction in the nanoparticle size to 44.65 nm after capping with chitosan. The highest mycelial growth reduction using fabricated Ch-AgNPs was 93% for *C. lunata* followed by 77% for *A. niger *and 66%* F. oxysporum at *(50 mg/L). Biosynthesis of AgNPs using A. atrobrunnea cell-free extract was successful. Capping with chitosan exhibited antifungal activity against fungal pathogens.

## 1 Introduction

Endophytic fungi reside within plant tissues without disrupting their physiological activities. A key role for these fungi is in synthesizing secondary metabolites in plants that act as antibacterial compounds and antioxidants and play various important roles (Rana et al., 2020). When exposed to metallic ions, these secondary metabolites can convert them into nano-sized particles, also known as nanoparticles (Sharma et al., 2022). Nanoparticles are significantly small particles that are extensively incorporated into medical, health care, and industrial fields because of their unique properties. These properties include the electronic, optical, thermal, and biological characteristics. Owing to their small size, these particles, when used in drugs, show strong adherence to target areas(Yu et al., 2013). Therefore, these drugs show an increased residence time and improved treatment efficiency. Increased residence time and adherence enable improved drug delivery efficacy resulting in successful therapy (Bahrami et al., 2017). AgNPs have shown an excellent ability to act as fungicidal agents, sensors, and anticancer agents (Saratale et al., 2019). In spite of these therapeutic benefits, the biological safety concerns surrounding their use, such as potential toxicity on cells, tissues, and organs, should be considered (Xu et al., 2020).

Therefore, extensive work is being done to fabricate AgNPs using various methods. AgNPs show strong adherence to the cell walls of microbial cell membranes and penetrate their cells, disrupting cellular structure by inducing the production of reactive oxygen species (ROS) and altering signal transduction (Siddiqi et al., 2018). In previous studies, it has been observed that AgNPs show efficient control against pathogenic microbes and are therefore widely used in agriculture and healthcare systems (Srivastava et al., 2021).Chemical techniques involving substances that reduce silver ions and stabilize nanoparticles are frequently employed to produce AgNPs(Natsuki et al., 2015). These substances are harmful and thus carry health-related risks and penetrate the food chain, which affects the ecosystem. Another approach for the fabrication of AgNPs is the biological approach where endophytic fungi and bacteria are employed in the formation of AgNPs (Srivastava et al., 2021). Studies have shown that biologically synthesized AgNPs exhibit increased abilities such as solubility, antimicrobial properties, stability, and improved yield. Moreover, the biological synthesis process is less toxic than the other processes and is a cost-effective method (Chernousova and Epple, 2013, Mukherjee et al., 2001). Different species, such as bacteria (Majumder et al., 2022), fungi (Seetharaman et al., 2018), plants (Velgosova & Veselovský, 2019), or algae (Velgosová & Mražíková, 2017) that can be utilized for nanoparticle formation, but endophytic fungi have been more effective at producing nanoparticles. AgNPs produced through biological synthesis are more stable because they are capped with biological molecules (Ishida et al., 2013). Moreover, biogenic synthesis is considered to be sustainable, clean, and economically important (Yan et al., 2018).

In recent years, endophytic fungi have also been studied for their ability to synthesize nanoparticles, including silver nanoparticles. Endophytic fungi have several advantages as a biological source of silver nanoparticles, including their abundance and diversity and their ability to produce nanoparticles with desirable physicochemical properties (Abdel-Azeem et al., 2020, Sandhu et al., 2017). Researchers have emphasized *Chaetomium* importance as an important genus in Ascomycota due to its numerous biological and biotechnological applications(Abdel-Azeem et al., 2020).

Chitosan is an important cationic polymer and a chitin derivative with various advantageous characteristics including biocompatibility, nontoxicity, and biodegradability. This natural polymer also exhibits antifungal activity, as well as mucoadhesive properties. Chitosan is mainly used to encapsulate of medicinal drugs because of its gelling properties and mucoadhesive characteristics. It is also used in the synthesis of oral drugs, as it plays a role in opening tight junctions of the mucosal membrane in the oral cavity and enhances absorption of the drug (Onyebuchi & Kavaz, 2019). Therefore, encapsulation of AgNPs with chitosan increases their efficiency, enhances drug delivery during therapeutic treatments, and is useful in the food and agricultural fields (Azevedo et al., 2014).

## 2 Material and methods

### 2.1 Chemicals and reagents

AgNO_3_ 99.9% (silver salt – the precursor of silver ions), low molecular weight chitosan ≤ 75% deacylated degree (Sigma-Aldrich CAT No. 448869), acetic acid glacial 99.8%, potato dextrose agar (PDA; Eur. Pharm., Scharlau Microbiology) and potato dextrose broth (PDB; Himedia, India). Sabouraud dextrose broth (SDB; Innovating Science), Czapek-dox broth (CDB), and glucose peptone yeast broth (GPYB) were prepared in the laboratory, Whatman filter paper No.1 and ultrapure deionized water.

### 2.2 Endophytic fungus source and maintenance

The endophytic fungus (ZS06) was previously isolated from *Ziziphus spina-christi* leaves, which were collected from Al-Baha region, Saudi Arabia, according to Abaya et al. (2021). The endophytic isolate was identified morphologically and by molecular techniques with the internal transcribed spacer (ITS): ITS1 forward (5′- TCCGTAGGTGAACCTGCGG-3′) and ITS 4 reverse (5′- TCCTCCGCTTATTGATATGC-3′) was used to amplify the ITS region. BLAST DNA sequences of the isolate were used for BLAST analysis (blast.ncbi.nlm.nih.gov) using default parameters, and sequencing data were analyzed against the nucleotide collection database. These were then compared using a BLAST search in GenBank. By employing Maximum Likelihood and Tamura-Nei models, the phylogenetic tree was inferred (Tamura & Nei, 1993). Identification at the taxonomic level was based on ≥ 99% ITS similarity. The GenBank accession number for the nucleotide sequence is OQ073876.

### 2.3 Production of fungal biomass

PDB (200 mL) was inoculated with endophytic isolate ZS06 spores for 7d at 25 ± 2 °C and agitated at 100 rpm. To eliminate any broth constituents, the mycelia were filtered through sterile Whatman filter paper No.1 and washed thrice with sterile deionized water. Fungal biomass (20 g) was mixed with 200 mL of sterile deionized water and agitated for 3d at 25 ± 2 °C. Then, filtered through sterile Whatman filter paper No.1 (Al-Khuzai et al., 2019, Netala et al., 2016).

### 2.4 Biosynthesis of AgNPs

The fungal filtrate (100 mL) and 100 mL of AgNO_3_ (1 mM) were mixed and incubated at 25 °C for 73 h at 150 rpm. Negative control (1 mM AgNO_3_) and positive control (CFF) were incubated in an experimental flask. The result was determined using (UV–vis) analysis.

### 2.5 Optimization of physicochemical factors in AgNPs production

#### 2.5.1 Effect of temperature

Sterile fungal biomass was added to deionized water (1:10 w/v) and incubated at diverse incubation temperature (25, 30, 35, 40, and 45 °C) for 3d. The results were determined using (UV–vis) analysis (Mizher, 2019).

#### 2.5.2 Effect of pH

Sterile fungal biomass was added to deionized water (1:10 w/v) at various pH values (5, 7, and 9) and agitated at 25 ± 2 °C for 24 h. The mycelium was then filtered through 0.22 µm filter paper and (1:1 v/v) of sterile filtrate and 1 mM AgNO_3_ and maintained at 25 °C until color change occurred. The results were determined using (UV–vis) analysis.

#### 2.5.3 Effect of molarity of AgNO_3_

The Sterile filtrate and AgNO_3_ (1:1 v/v) were mixed at different molarities (1, 2, 3, 4, and 5 mM) and agitated at 25 °C for 24 h. The results were determined using (UV–vis) analysis.

#### 2.5.4 Effect of culture media

To understand the possible impact of culture media on fungal activity and biosynthesis of NPs, four different media were used, namely PDB, GPYB, CDB, and Sabouraud broth (SAB). All the above media were evaluated for optimum NPs synthesis. Fungal spores were cultivated for 7d in (250 mL) of the test media in each flask. AgNPs were synthesized as described above and then subjected to UV–Vis analysis.

### 2.6 Encapsulating AgNPs with chitosan

Chitosan (15.0 g/L) was dissolved in glacial acetic acid (1.0%) using magnetic stirrer until dissolved and left overnight (El-Sherbiny & Sedki, 2019). The suspension of AgNPs was mixed with chitosan in dropwise manner (1:2 v/v). Then, exposed to microwave according to Raza et al. (2021) technique followed by cooling before examination then preserved at 4 °C.

#### 2.6.1 Characterization of AgNPs and Ch-AgNPs

Absorption spectral analysis of Ch-AgNPs was measured using a Varian Caryâ 50 UV–vis spectrophotometer in room temperature at the wavelength between 300 and 600 nm at a resolution of 1 nm and deionized water purified by Milli-Q purification system (Millipore Corporate, Billerica, MA) which used as a blank (Birla et al., 2013). FTIR spectroscopy was performed using (Thermo Fisher, Nicolet iS10) in the range of 600–4000 cm^−1^ at a resolution of 4 cm^−1^ (Cui et al., 2022). Zeta-potential and particle size distribution were estimated following Ma et al. (2016)’s protocol using a Zetasizer Ver.7.13 (Malvern-Instruments Ltd. Malvern, UK). The AgNPs and Ch-AgNPs were subjected to FE-SEM using (FE-SEM, JEOL, JSM- IT700HR, Tokyo, Japan), which provides topographic details of the surface or entire AgNPs and Ch-AgNPs samples. The average size of fabricated nanoparticles was estimated via ImageJ and Origin software.

### 2.7 Antimicrobial activity of Ch-AgNPs

#### 2.7.1 Mycelial growth inhibition (MGI)

The antifungal effect of Ch-AgNPs was investigated by inhibiting the radial growth of *F. oxysporum, C. lunata,* and *A. niger* using the food poisoning technique. A 6 mm mycelium disc was cut from the margins of a 5-d-old fungal culture and transferred to seeded PDA plates with Ch-AgNPs (6.25, 12.5, 25, and 50 mg/L) in triplicate and incubated for 5d at 25 °C. The protocol applied on PDA without AgNPs as controls. The MGI ratio was determined using the following equation (da Silva Bomfim et al., 2015);

$$MGI\left(\%\right)=\left[\mathrm{dc}-dt\right]/dc\times 100$$

The mean fungal growth diameter dt (mm) is measured for each group treated with Ch-AgNPs, whereas dc (mm) is measured for each group not treated with Ch-AgNPs.

### 2.8 Statistical analysis

All data expressed as means ± SD. A multivariate analysis of variance (ANOVA) was used to compare the differences in the means of the various groups using (GraphPad version 9.4.1, San Diego, CA, USA). Statistical significance for all tests was set at *p* < 0.05, *p* < 0.01 and *p* < 0.001.

## 3 Results

### 3.1 Endophytic fungus isolate ZS06 identification

**Fig. 1:**
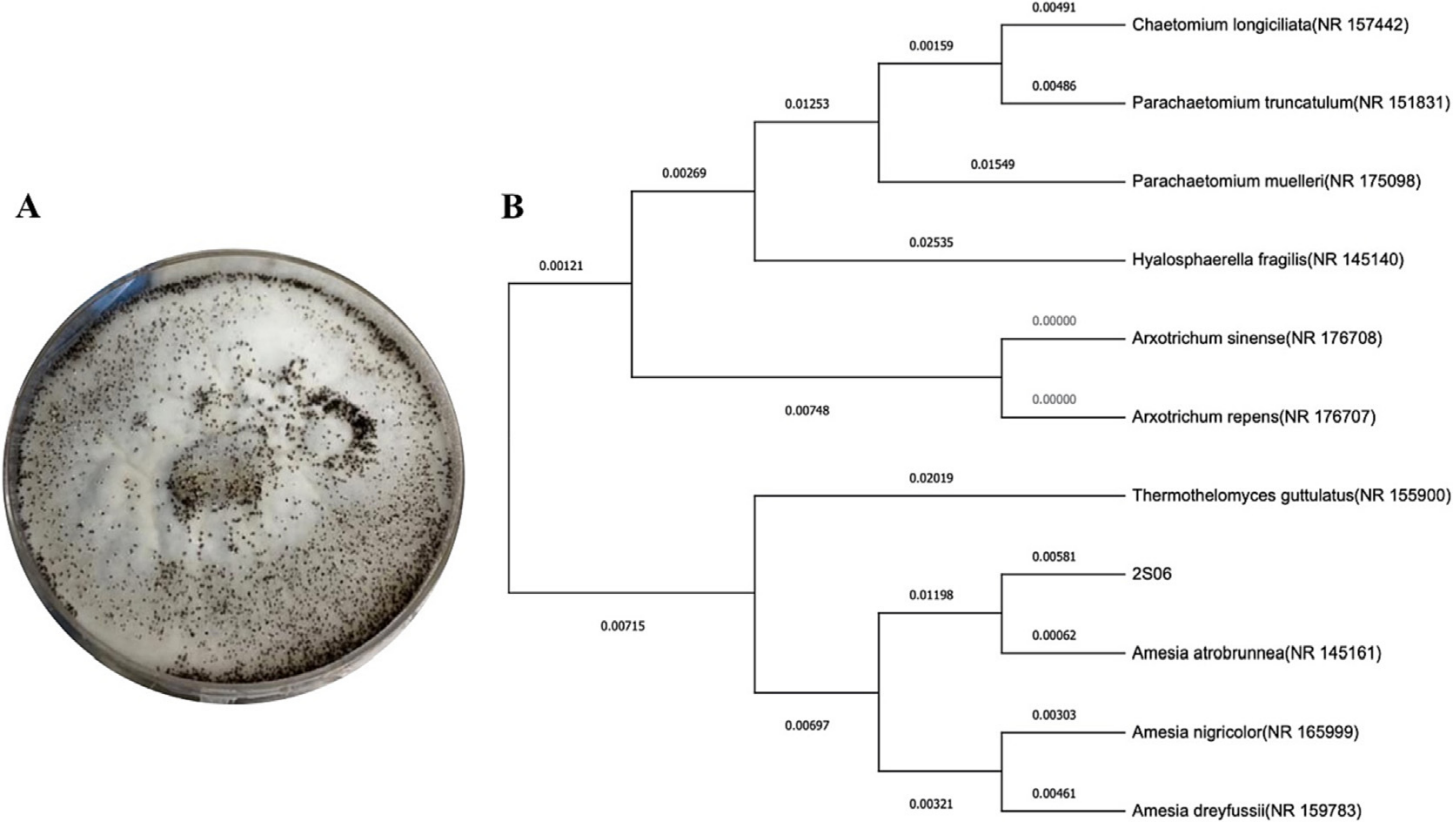
Endophytic fungus isolate *A. atrobrunnea* (ZS06) on PDA medium after 7d at 25 °C (A). neighbor-joining tree based on an alignment of the ITS gene sequence, which demonstrated the connection between the results from the ZS06 gene and those from the NCBI database. (B). *A. atrobrunnea* and ZS06′s ITS gene sequence had a significant degree of similarity.

The endophytic fungus isolate ZS06 was purified, and its appearance on PDA medium was characterized by woolly white hyphae on the back and numerous dark-brown perithecia (Fig. 1A). Blastn, pairwise, and multiple sequence alignment showed 99.36% identity with *A. atrobrunnea* strains (Basionym: *Chaetomium atrobrunneum*), and given Accession Number OQ073876 in NCBI GenBank. The phylogenetic tree was constructed using Maximum Likelihood, and the percentage of trees in which the related taxa clustered together is indicated next to the branches (Fig. 1B).

### 3.2 Biosynthesis of AgNPs from *A. atrobrunnea*

**Fig. 2:**
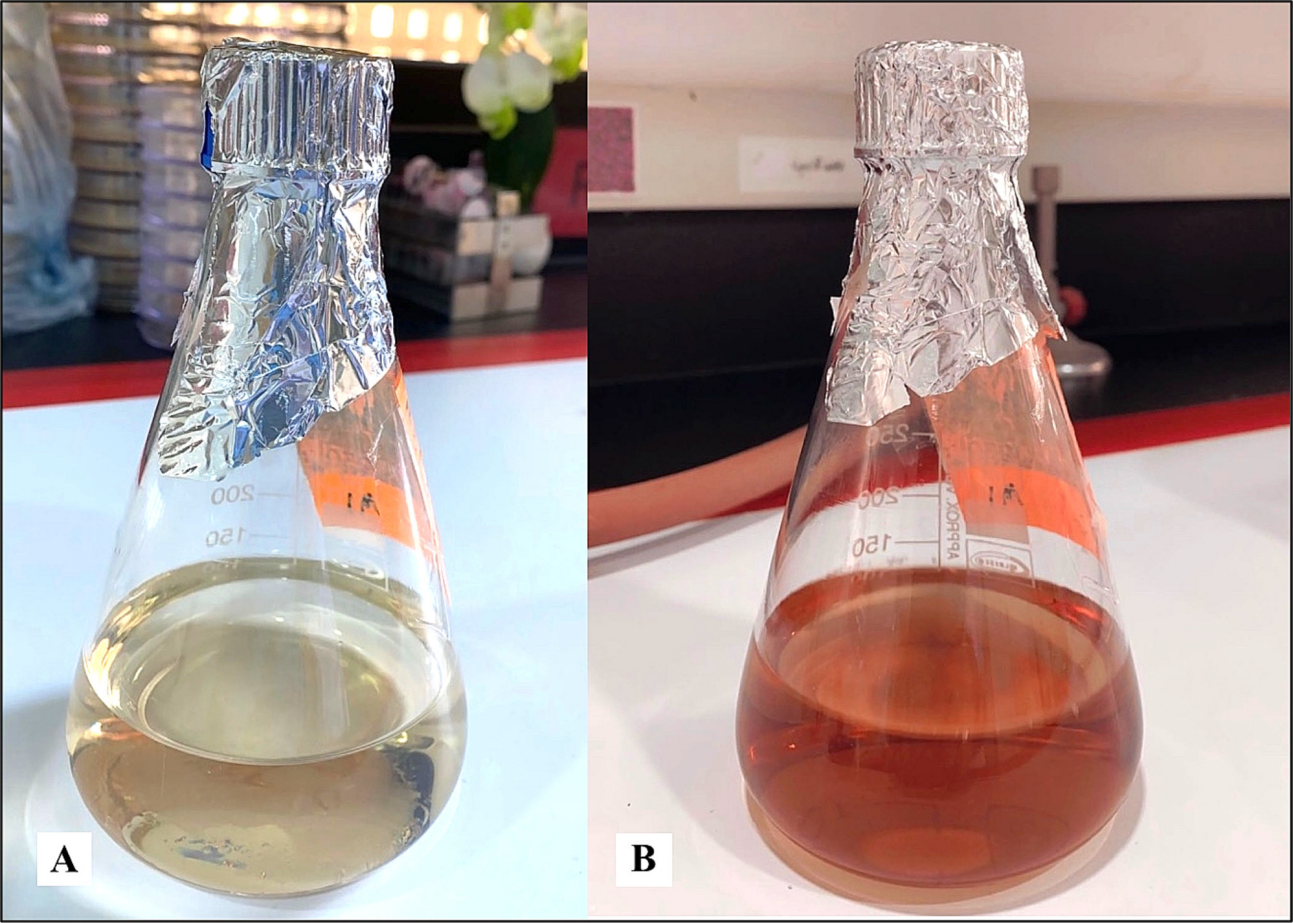
Culture flasks containing *A. atrobrunnea* ZS06 cell-free filtrate (A) and *A. atrobrunnea* ZS06 cell-free filtrate after addition of 1 mM AgNO_3_(B).

**Fig. 3:**
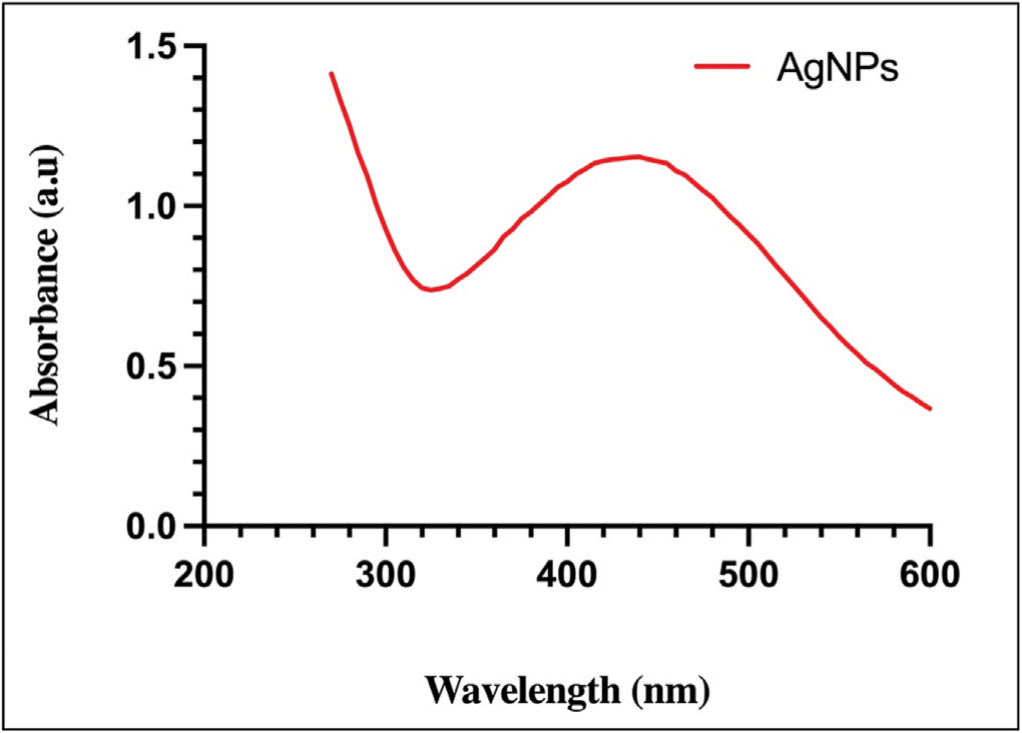
UV–vis spectrum of *A. atrobrunnea* AgNPs.

After 73 h, the color of the filtrate changed from pale yellow to honey brown, as depicted in (Fig. 2A and B). The formation was confirmed via UV–vis spectroscopy, with the maximum absorption of AgNPs at 435 nm (Fig. 3).

### 3.3 Optimization the conditions for biosynthesis of AgNPs

#### 3.3.1 Effect of temperature

**Fig. 4:**
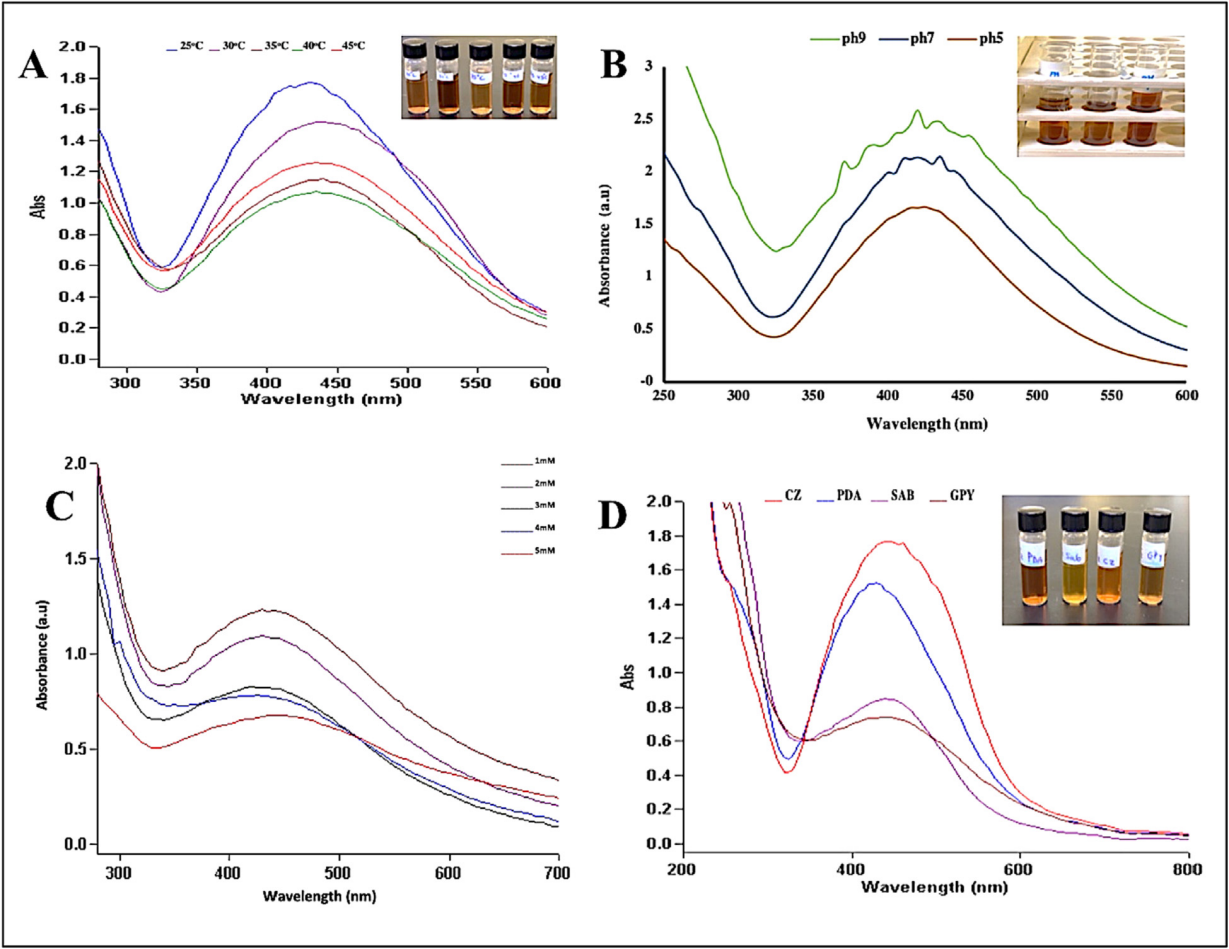
UV–vis spectra of the AgNPs from *A. atrobrunnea* filtrate at different (25, 30, 35, 40, and 45 °C) (A), at pH (9, 7, and 5) (B), at AgNO_3_ concentrations (1, 2, 3, 4, and 5 mM) (C), and with different media (CDB, PDB, SAB, and GPYB) (D).

By adjusting the fungal culture incubation temperature, it is possible to alter the fungus’ metabolism to produce nanoparticles with desirable properties, such as a particular size and form (Zielonka and Klimek-Ochab, 2017). The CFF resulting from biomass incubated at (25, 30, 35, 40, and 45 °C) showed a reduction of AgNPs that ended after 73 h incubation with AgNO_3_ (Fig. 4A). Among these, the stability and excellent nanoparticle production were demonstrated at a temperature range of 25–35 °C. Increasing fungal incubation temperatures to 40–45 °C showed the rapid formation of AgNPs; however, agglomeration accrued after one week.

#### 3.3.2 Effect of pH

The pale-yellow CFF turned dark brown after the addition of AgNO_3_ (Fig. 4B). The color change was immediate at pH 9 and 5, but they were not stable and agglomeration of the solution occurred 2d after the formation of nanoparticles. At a pH 7, AgNPs were synthesized efficiently and were stable. Thus, this pH value was chosen for future investigation.

#### 3.3.3 Effect of AgNO_3_ concentration

The concentration of 1 mM showed maximum absorbance (1.152 a.u.) at 435 nm, indicating the efficient production of AgNPs (Fig. 4C). The graph shows that the yield of AgNPs gradually decreased with increasing AgNO_3_ concentration. The highest concentration (5 mM) exhibited instability, with aggregation and precipitation at the bottom.

#### 3.3.4 Effect of culture media

The effects of different culture media on AgNPs biosynthesis and enzyme secretion by the fungus were tested. Four cell-free filtrates were used for AgNPs’ biosynthesis obtained from biomass grown on (PDB, GPYB, CDB, and SDB). This is evident from (Fig. 4D). The highest AgNPs production was recorded in CDB medium (1.832 a.u.) at 435 nm, followed by PDB, SDB, and GPYB.

### 3.4 Capping AgNPs with chitosan polymer

A polycationic biopolymer (chitosan) was used with the previously synthesized AgNPs to fabricate polymer-based nanocomposites, Ch-AgNPs, using microwave irradiation. For the formation of Ch-AgNPs, low-molecular-weight chitosan was used. A higher-molecular-weight biopolymer would have made the nanocomposite system larger, exceeding the nanoscale range.

### 3.5 Characterization

#### 3.5.1 UV–vis of Ch-AgNPs

**Fig. 5:**
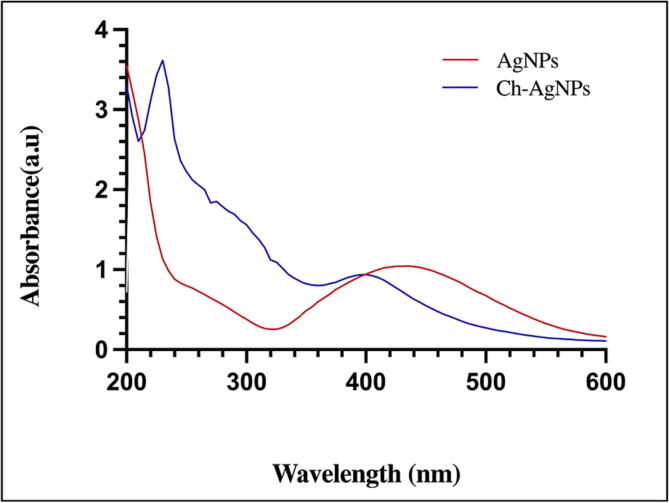
UV–vis spectra of *A. atrobrunnea’* AgNPs and Ch-AgNPs.

Maximum absorbance at 430 nm revealed the presence of AgNPs (Fig. 5). For the Ch-AgNPs nanocomposites, two different clean peaks were detected at 405 nm and 230 nm, confirming the development of chitosan capped AgNPs.

#### 3.5.2 FTIR spectroscopy analysis

**Fig. 6:**
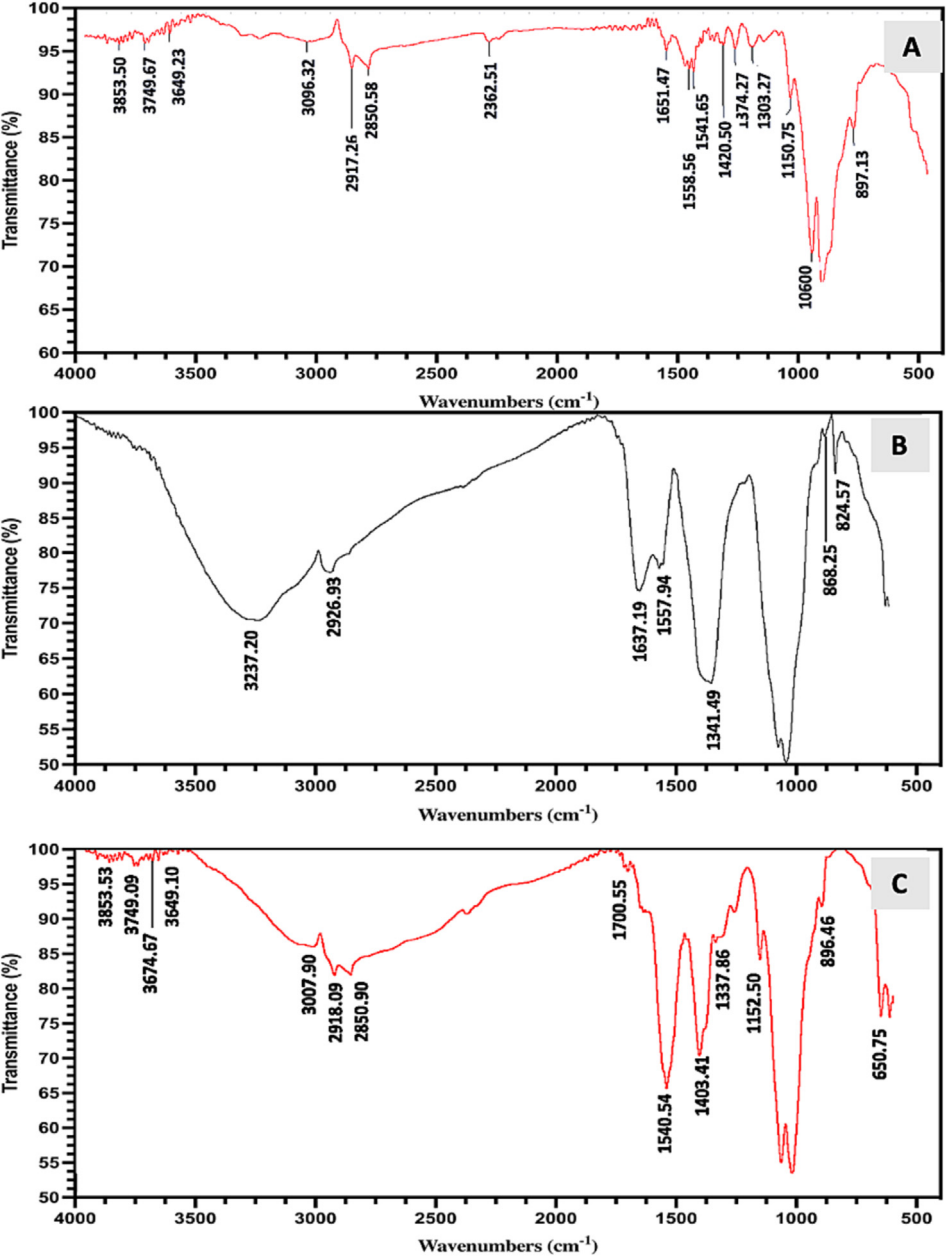
FTIR spectrum of (A) Chitosan, and (B) AgNPs synthesized by *A. atrobrunnea* (C) Ch-AgNPs.

In the FTIR spectrum of chitosan in Fig. 6**A**, peaks within 3749.67–3853.50 cm^−1^ and at 3649.23 and 3096.32 cm^−1^ are assigned to free O–H *stretching*, free N–H *stretching* + mutual OH•••NH hydrogen-bonding, and hydrogen bonded N–H *stretching* vibration, respectively. The *symmetric* –CH_2_– *stretching* and *asymmetric* –CH_2_– *stretching* were recorded at 2850.58 and 2917.26 cm^−1^, respectively. Functional groups in AgNPs and Ch-AgNPs were detected using FTIR spectroscopy. AgNPs FTIR spectra is presented in Fig. 6**B**, the peaks at 3237.20 and 2926.46 cm^−1^ show N–H s*tretching* and O–H s*tretching* vibrations of primary amines and –CH2– *stretching*, respectively. The protein in the fungal extract used for the reduction of nanoparticles was confirmed by the amide-specific peaks at 1636.53, 1558.99, and 1336.90 cm^−1^. Furthermore, FTIR spectrum of Ch-AgNPs **(**Fig. 6**C)** comprises the characteristic peaks of *A. atrobrunnea* AgNPs and peaks of the capping agent chitosan. Importantly, the strong interactions between the modified AgNPs and chitosan were confirmed by the significant shifting of the peak position from the original values. Capping of chitosan is confirmed from the peaks within 1018.79–1152.50 and at 1403.41, and 2850.90 cm^−1^ for the vibrations of –CH_2_–O–CH_2_–, –CH_2_OH (side chain), C–OH bending vibrations of OH, CH in ring, and –CH_2_– str. for the pyranose ring of chitosan. Interactions between AgNPs and chitosan through –CONH- functionalities were confirmed by the altered peaks at 1540.54 cm^−1^ and 1700.55/ 1620 cm^−1^ for NH-*bending* vibration in the amide group and carbonyl (>C = O) *stretching* vibrations in the amide linkages of proteins, respectively.

#### 3.5.3 Zeta-potential

**Fig. 7:**
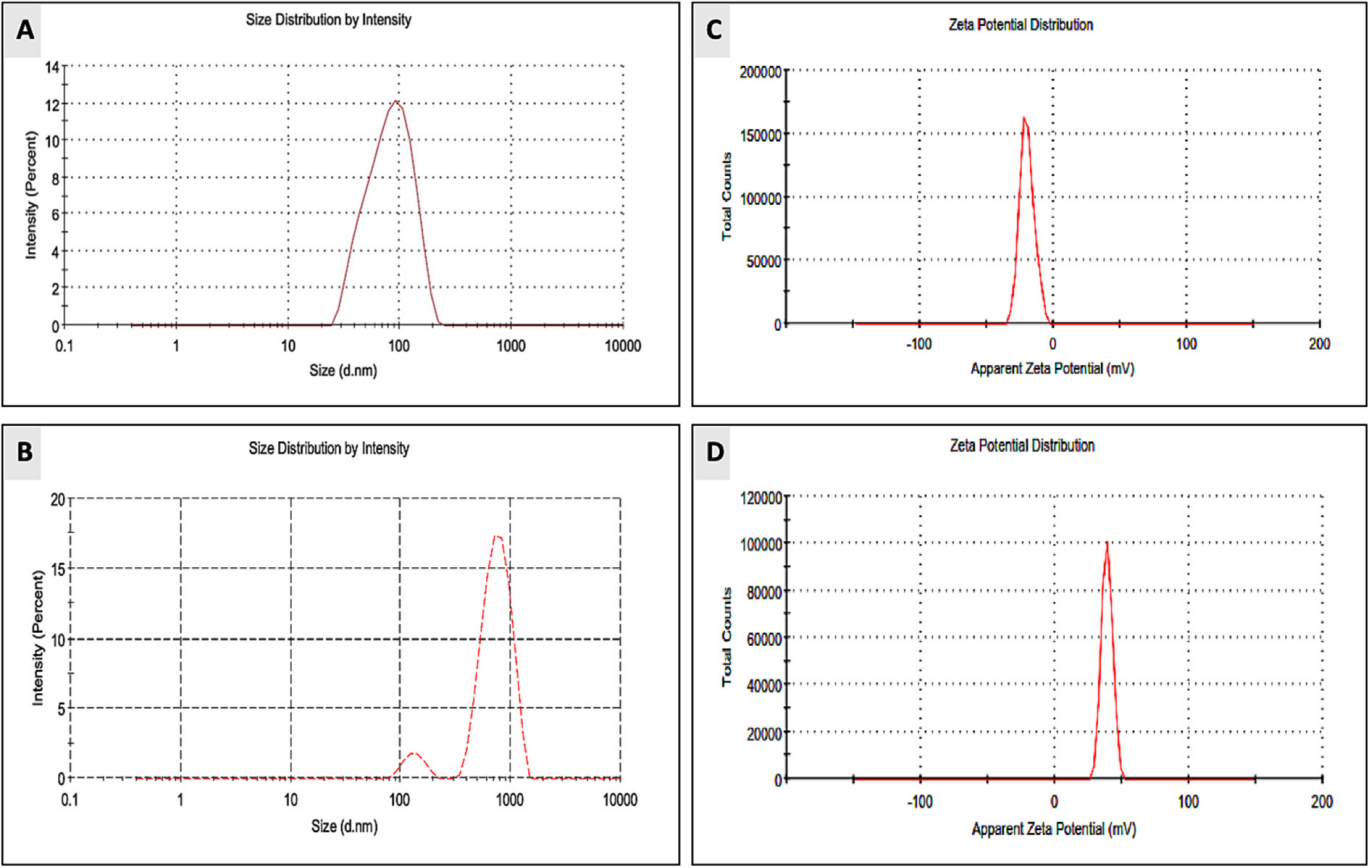
Average size distribution and surface charge of AgNPs (A, B) average size distribution and surface charge of Ch-AgNPs (C) and (D) Z-potential values of AgNPs and Ch-AgNPs estimated using DLS.

The size of the zeta potential reflected the nanoparticles potential for stability. Once all particles have a strong positively or negatively zeta potential, they oppose one another, resulting in stable dispersion. The Z-size distributions of the AgNPs and AgNPs after capping with chitosan **(**Fig. 7**A and C)** showed a narrow size distribution curve with PdI 0.3, 0.6 respectively. The results for the Z-potential AgNPs and Ch-AgNPs nanocomposites are shown in **(**Fig. 7**B and D)**. The polydisperse nature is a result of their extremely negative zeta potential, which precludes the development of agglomerates and leads to their stability. The results are summarized in **(**Table. 1**)** indicates that all AgNPs were transformed from anionic to cationic particles. Furthermore, nanoparticle stability was significantly enhanced after capping by increasing the surface charge of NPs.

**Table 1:** A comparison between zeta potential results and of AgNPs and Ch-AgNPs.

**Sample**	**Z-potential**	**PDI**	**Z-size (d.nm)**
**AgNPs**	−19.7	0.341	339
			43.39
**Ch-AgNPs**	38.9	0.66	763.7
			156.1

#### 3.5.4 FE-SEM

**Fig. 8:**
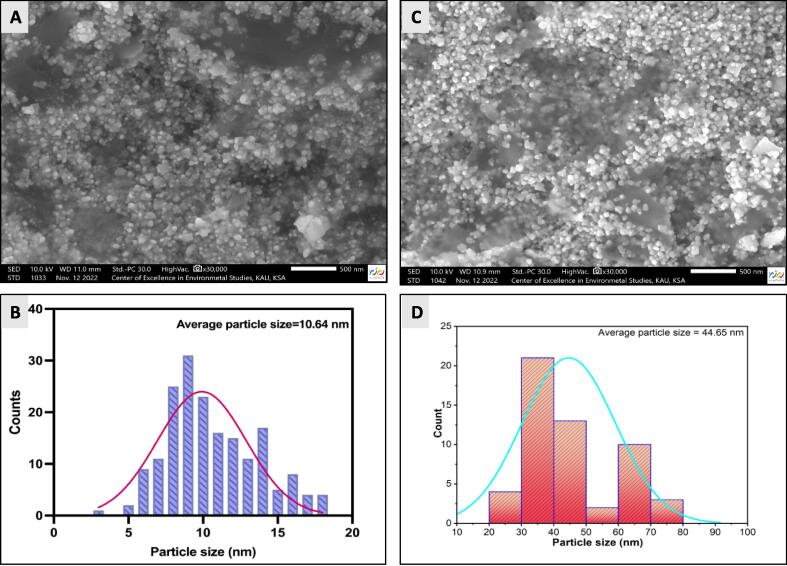
FE-SEM images of AgNPs of *A. atrobrunnea* at a magnification of 30.000X magnification (A) Ch-AgNPs after capping with chitosan at a magnification of 30.000X magnification (B) Histogram for size distribution of AgNPs (C), and Ch-AgNPs (D).

FE-SEM was utilized to examine the morphology of *A. atrobrunnea*’s NPs. Based on the findings, the AgNPs without chitosan possessed an average particle size of 10.64 nm and were visible on the surface as spherical white spots. A small percentage of the nanoparticles also underwent aggregation, resulting in a larger size of nanoparticles. FE-SEM characterization demonstrates AgNPs surface morphology before and after chitosan coating **(**Fig. 8**A and B).** FE-SEM images revealed that the average particle size of the Ch-AgNPs was 44.65 nm calculated using ImageJ software. Also, FE-SEM was utilized to improve evaluate the form of AgNPs without coating, which showed a smoother, more uniform surface with less roughness than that of Ch-AgNPs. Numerous luminous brilliant spots were observed in the high-magnification image of the AgNPs in Fig. 8**A.**

In Fig. 8**B, a** thin chitosan coating encapsulates AgNPs and greatly occupies the spaces between nanoparticles that was visible. Fig. 8**C and D** represents the size distribution of AgNPs without coating with chitosan and Ch-AgNPs after coating with chitosan, based on ImageJ analysis.

### 3.6 Antifungal activity

#### 3.6.1 MGI

**Fig. 9:**
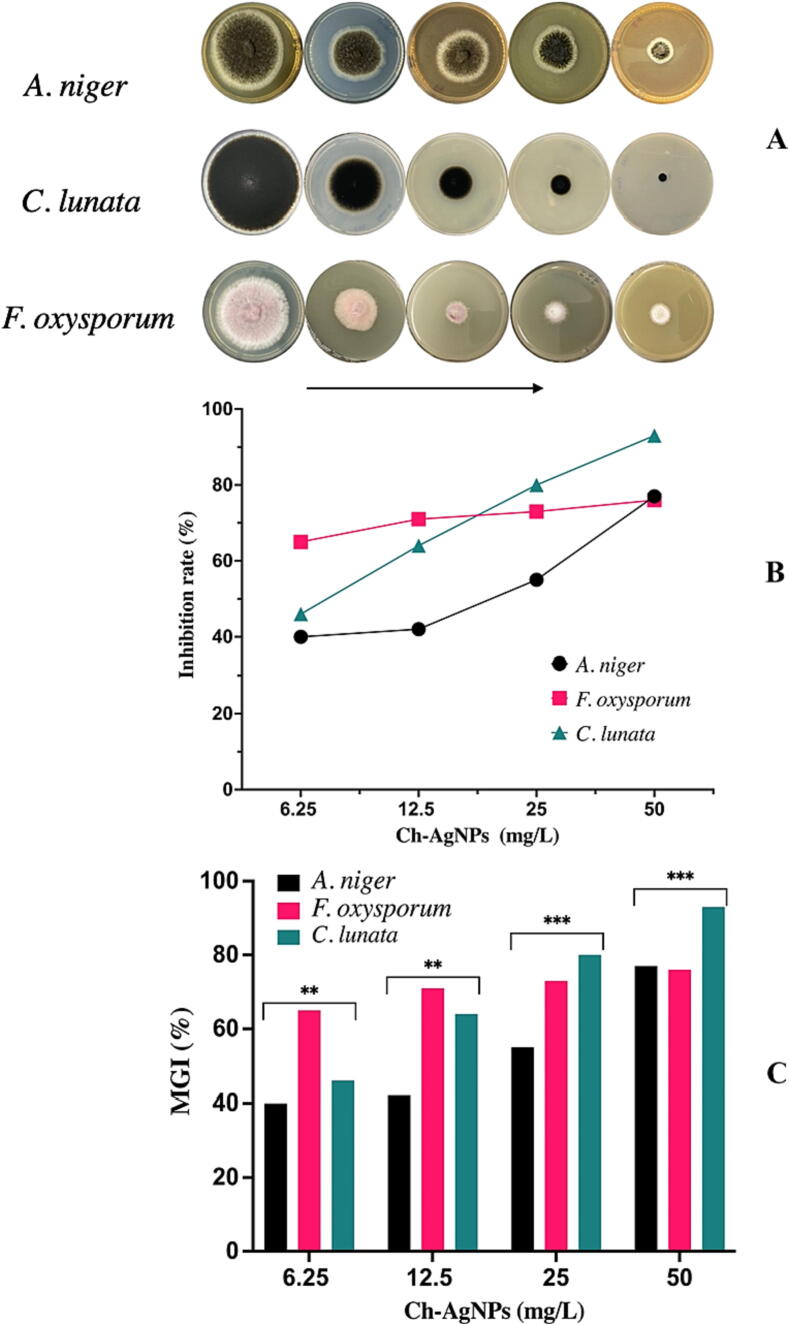
The mycelium growth inhibition of *A. niger*, *C. lunata,* and *F. oxysporum* on PDA plates with different concentrations of Ch-AgNPs from left to right (0, 6.25, 12.5, 25, and 50 mg/L) (A), The correlation between Ch-AgNPs concentration and the MGI rate (B), MGI% of tested fungi with different concentration Ch-AgNPs (C). All values shown in the graph are means of triplicates (±SD). (*) indicates significant differences (P < 0.05) in comparison to their controls respectively.

The anti-fungal activity of Ch-AgNPs were investigated against *F. oxysporum*, *C. lunata*, and *A. niger* **(**Fig. 9**A).** The mean colony dimeter calculated in (mm) and mycelium growth inhibition (%) represented in (Table 2). At the concentration of Ch-AgNPs was 50 mg/L, the inhibition rate of Ch-AgNPs to *C. lunata* (93%) which was higher than that of *A. niger* (77%), and *F. oxysporum* (76%). While 25 mg/L inhibited the mycelial growth by (80%, 73% and 55%) for *C. lunata, F. oxysporum* and *A. niger* respectively. Also, 12.5 mg/L inhibited the mycelial growth by (71%, 64% and 42%) for*, F. oxysporum*, *C. lunata* and *A. niger* respectively. The lowest concentration 6.25 mg/L showed minimum inhibition rate (65%, 46% and 40%) for *F. oxysporum*, *C. lunata* and *A. niger* respectively. Ch-AgNPs exhibited concentration-dependent mycelial inhibition **(**Fig. 9**B),** whereas anti-fungal activity increased with the increase of Ch-AgNPs concentration. The results are presented in **(**Fig. 9**C),** indicating that *F. oxysporum* is more sensitive to Ch-AgNPs than *C. lunata* and *A. niger.*

**Table 2:** Inhibition MGI of Ch-AgNPs against tested fungi in vitro.

**Ch-AgNPs (mg/L)**	***A. niger***		***C. lunata***		***F. oxysporum***	
	Colony dimeter (mm)	MGI (%)	Colony dimeter (mm)	MGI (%)	Colony dimeter (mm)	MGI (%)
Control	82 ± 2.64	0	83 ± 2.64	0	90 ± 2.88	0
6.25	49.6 ± 0.57	40^**^	45.1 ± 0.85	46^**^	31.6 ± 0.57	65^**^
12.5	47.7 ± 0.68	42^**^	29.6 ± 1.03	64^**^	25.8 ± 2.02	71^**^
25	37.3 ± 0.57*	55^***^	16.83 ± 0.76*	80^***^	24.6 ± 0.57*	73^***^
50	18.6 ± 1.15^***^	77^***^	6.2 ± 0.5^***^	93^***^	22 ± 0.3^***^	76^***^

*Values are the average of three individual experiments. Data are presented as mean ± SD. Means followed by (*) in the same column differ significantly (*p* = 0.001) according to Tukey’s test.*=*p <* 0.05*,* **=*p <* 0.01,*** =*p <* 0.001.

## 4 Discussion

The importance of this study is that biosynthesized silver nanoparticles from endophytic fungus *A. atrobrunnea* filtrate encapsulated with chitosan have the ability to inhibit the growth of phytopathogenic fungi. Also, the biosynthesis of Ch-AgNPs described in this study is cost-effective, environmentally friendly also of importance. Since Ch-AgNPs have been demonstrated to be antimicrobial, they may have anti-fungal potential on phyto-pathogenic fungi (Mondéjar-López et al., 2023, Pham et al., 2018, Shehabeldine et al., 2022). The honey-brown color, which appeared after *A. atrobrunnea* cell-free filtrate was mixed with AgNO_3_ is proof of the bio-reduction of silver ions and the formation of AgNPs. Numerous reports in the scientific literature describe the production of various shades of color, ranging from yellow and honey brown to dark brown, in AgNPs (Cui et al., 2022, Qian et al., 2013, Singh et al., 2017). The UV–vis spectroscopy used to confirm the formation AgNPs and wavelengths due to the particles’ surface plasmon resonance (Vahabi et al., 2011). UV–vis absorbance of AgNPs may vary according on the microbe utilized, with values between 400 and 450 nm (Elamawi et al., 2018). The result agrees with the maximum absorbance between 420 and 450 nm of AgNPs synthesized by *Chaetomium globosum* and *Trichoderma viride* (Abdel-Azeem et al., 2020, Abu-Elsaoud et al., 2015, Madbouly et al., 2017).

The stability and excellent nanoparticle production were demonstrated at a temperature range of 25–35 °C. These results showed similarity with the results obtained from extracellular biosynthesis of AgNPs using *Chrysosporium tropicum*, *F. oxysporum* (Soni & Prakash, 2011) and *A. niger* (Mizher, 2019). The optimum pH for various microorganisms to biosynthesize AgNPs varies considerably. An efficient and stable AgNPs was formed at pH 7 while, the formation at pH 9 and 5, were high but not stable and agglomerated after 2d. In agreement with Birla et al. (2013), *F. oxysporum* produced the highest nanoparticles at alkaline pH values (9–11). Alkaline pH results in greater synthesis success because metal ions and protons compete more aggressively for bonds with negatively charged regions (Sintubin et al., 2009).While in Husseiny et al. (2015) study, neutral pH values decreased the production of AgNPs and acidic pH values caused aggregation pH (3–5). Depending on the concentration of protons in the reaction medium, the conformation of the nitrate reductase enzyme can change, altering the nanoparticle morphology and size (Nayak et al., 2011).

It has been found that lower amount of metal salts concentrations led to reduce nanoparticles size and better dispersion in some cases (Phanjom & Ahmed, 2017). This was mainly caused by the limited number of functional groups for the biosynthesis reaction in CFF (Guilger-Casagrande & Lima, 2019). Good agreement was found when comparing our results from this work with published data for biosynthesis using *A. niger* and *Penicillium purpurogenum* (Mizher, 2019, Nayak et al., 2011).

Different culture media compositions are one of the parameters that affects the types of metabolites and proteins excreted by microbial cells. Therefore, it is reasonable to expect different production intensities of AgNPs in different media (Anil Kumar et al., 2007). In this study, the highest AgNPs production was recorded in CDB medium. This may be owing to the presence of substances in CDB that promote better development of *A. atrobrunnea* (Basionym: *Chaetomium atrobrunneum*) and help in produce more silver ion reducing agents. In this respect, Abo-Elmagd (2014) found that altering carbon and nitrogen sources enhanced *Chaetomium madrasenses* bioactive properties. Sucrose in CDB was favorable for antioxidant activity of the fungus when compared to glucose was used in other media while sodium nitrate is more favorable as a carbon source compared to peptone and yeast extracts. That might enhance development AgNPs production of *A. atrobrunnea* by enhancing the bioactive properties.

Similar results obtained by optimizing AgNPs condition from *Chaetomium globosum* which found 1.5 mM AgNO_3_ with pH between 6 and 8, and 25 °C incubation temperature were the optimum conditions for highest production (Abu-Elsaoud & Abdel-Azeem, 2020).

The use of microwaves generates monodisperse Ch-AgNPs with perfect amalgamation (Zhang et al., 2007). It is possible to enhance the characteristic morphological structure and overall charge pattern of the AgNPs because of the outer coating of chitosan, without altering the physicochemical properties of the AgNPs (Raza et al., 2021). The Ch-AgNPs maximum peaks were recorded two distinct sharp peaks were recorded at 405 nm and 230 nm in agreement with previous studies (Peng et al., 2017, Zhang et al., 2014).

FTIR peaks at 1651.47, 1541.65/ 1558.56, and 1456 cm^−1^ were attributed to the > C = O *stretching* of the amide group (amide-I band), NH-*bending* vibration of the amide group, and C–N *stretching*, respectively (Biao et al., 2017). These peaks are assigned to carbonyl stretching vibrations (1636.53 cm^−1^), NH-bending vibration (1558.99 cm^−1^), and C-N stretching vibrations (1336.90 cm^−1^) in amide groups of fungal proteins. These findings are comparable with the results of published studies (Bagur et al., 2022, Ballottin et al., 2016, Singh et al., 2017). After binding with chitosan, a significant reduction in mutual OH•••NH hydrogen bonding was noted from the reduction in the intensity of the characteristic peaks (Asghar et al., 2020, Raza et al., 2021).

However, Z-potential of AgNPs (-19.7) and Ch-AgNPs (+38.9) confirm that all AgNPs were transformed from cationic to anionic particles. Z-potential between −10 and + 10 mV were considered to be almost neutral and with low stability nanoparticles. While, strongly stable cationic and anionic particles are nanoparticles with Z-potential more than + 30 mV or less than −30 mV, respectively (Bhattacharjee, 2016, Khoshnevisan and Barkhi, 2015, Mourdikoudis et al., 2018).

Chitosan was used to create nanoparticles with an average size in nanoscale (≤100 nm) that were spherical in shape. This was attributed to the presence of chitosan’s macromolecules which stabilizes and prevents nanoparticles in the reaction mixture from aggregating. Chitosan has a large number of binding or functional sites that can be used to cap AgNPs through non-covalent interactions (Koo et al., 2023). In good agreement was found when comparing results of FE-SEM from this work with published data on chitosan-capped AgNPs (Raza et al., 2021).

Antifungal activity of Ch-AgNPs showed significant antifungal activity against *F. oxysporum*, *C. lunata*, and *A. niger* in solid media. Compared to chitosan nanoparticle-encapsulated biomolecules, the results were comparable with published data that focused on the use of these nanocomposites to control fungal pathogens, including postharvest fungi such as *A. niger* and *A. flavus* and preharvest fungi that infect plants and crops such as *F. oxysporum, Rhizopus stolonifera,* and *Botrytis cinereal* (Hasheminejad et al., 2019, Mondéjar-López et al., 2022, Ren et al., 2021, Salgado-Cruz and et al., 2021). Similar results were obtained using Ch-AgNPs against *F. Oxysporum,* and another *Aspergilli* sp*.* Chitosan silver nanocomposites showed a higher inhibition of fungal radial growth than chitosan nanoparticles at all tested concentrations (Dananjaya et al., 2017). This may be caused by the presence of negative charges in fungal cell membranes, and chitosan coating may enhance the anti-fungal potential of bio-AgNPs (Fan et al., 2018).

## 5 Conclusion

To the best of our knowledge, endophytic fungus *A. atrobrunnea* extract was used for the first time to reduce silver ions to AgNPs in the present study. The results demonstrated the potential of fungal proteins in the reduction of AgNPs and confirmed that chitosan was successfully incorporated with AgNPs as a capping agent. Ch-AgNPs (44.65 nm) significantly inhibited the radial growth of the fungal pathogens *F. oxysporum, C. lunata,* and *A. niger.* The method described in this study is optimal for use against fungal resistance to chemical fungicides in agriculture applications, considering their safety to the environment. Thus, the prepared Ch-AgNPs can be used for the development of the medicinal industry.

## Declaration of Competing Interest

The authors declare that they have no known competing financial interests or personal relationships that could have appeared to influence the work reported in this paper.
